# Role of qSOFA in predicting mortality of pneumonia: A systematic review and meta-analysis

**DOI:** 10.1097/MD.0000000000012634

**Published:** 2018-10-05

**Authors:** Jianjun Jiang, Jin Yang, Yongmei Jin, Jiyu Cao, Youjin Lu

**Affiliations:** a Department of Respiratory Medicine, The Second Affiliated Hospital of Anhui Medical University; b The Teaching Center for Preventive Medicine, School of Public Health, Anhui Medical University, Hefei, China.

**Keywords:** mortality, pneumonia, prognosis, qSOFA

## Abstract

**Background:**

The concept of sepsis was redefined recently, and a new screening system termed the quick Sequential Organ Failure Assessment (qSOFA) was recommended for identifying infected patients at high risk for death. However, the predictive value of qSOFA for mortality in patients with pneumonia remains unclear. Thus, we performed a meta-analysis with the aim of determining the prognostic value of qSOFA in predicting mortality in patients with pneumonia.

**Methods:**

Embase, Google Scholar, and PubMed (up to March 2018) were searched for related articles. We constructed a 2 × 2 contingency table according to mortality and qSOFA scores (<2 and ≥2) in patients with pneumonia. Two investigators independently extracted data and assessed study eligibility. A bivariate meta-analysis model was used to determine the prognostic value of qSOFA in predicting mortality. *I*^2^ index and *Q*-test were used to assess heterogeneity.

**Results:**

Six studies with 17,868 patients were included. A qSOFA score ≥2 was related to a higher risk for death in patients with pneumonia, with a pooled risk ratio (RR) was 3.35 (95% CI, 2.24–5.01) using a random-effects model (*I*^2^ = 89.4%). The pooled sensitivity and specificity of a qSOFA score ≥2 to predict mortality in patients with pneumonia were 0.43 (95% CI, 0.33–0.53) and 0.86 (95% CI, 0.76–0.92), respectively. The diagnostic OR was 4 (95% CI, 3–6). The area under the summary receiver operator characteristic (SROC) curve was 0.67 (95% CI, 0.63–0.71). When we calculated the community-acquired pneumonia (CAP) subgroup, the pooled sensitivity and specificity were 0.36 (95% CI, 0.26–0.48) and 0.91 (95% CI, 0.84–0.95), respectively. The area under the SROC curve was 0.70 (95% CI, 0.66–0.74).

**Conclusions:**

A qSOFA score ≥2 is strongly associated with mortality in patients with pneumonia, but the poor sensitivity of qSOFA may have limitations in the early identification of mortality in patients with pneumonia.

## 1 Introduction

Pneumonia is a common cause for hospitalization and mortality worldwide,^[1]^ approximately 20% of community-acquired pneumonia (CAP) in adult patients require hospitalization and has a mortality rate of 30% to 50%.^[2]^ Despite ongoing advances in life-support measures and antimicrobial therapy, pneumonia is still a significant infection burden worldwide, and it is often complicated by sepsis.^[3–5]^ Furthermore, the mortality rate in patients with pneumonia-associated sepsis/septic shock increased to over 50%.^[6]^ However, early recognition of sepsis is the basis for guiding therapy, improving outcomes, and reducing costs.^[7,8]^ Therefore, to decrease the mortality rate due to pneumonia, it is important to accurately assess the severity of pneumonia during the initial assessment and then determine whether aggressive therapy and close monitoring are more appropriate than conservative therapy.

Several pneumonia severity scales have been developed to distinguish patients at high risk of death and support therapeutic decisions.^[9]^ Among these scales, CURB-65 (confusion, urea nitrogen, respiratory rate, blood pressure, age ≥ 65 years) and pneumonia severity index (PSI) are well-validated scales to support clinical decision-making and CAP prognosis.^[9,10]^ Different simplifications of CURB-65 are available, including CRB-65 (confusion, respiratory rate, blood pressure, age ≥ 65 years) and CRB^[11]^ (confusion, respiratory rate, blood pressure), to facilitate the risk stratification process as these simplified scales do not require blood urea measurements.^[12]^

For sepsis, the definition of this syndrome was recently modified, and a new criteria termed the quick Sequential Organ Failure Assessment (qSOFA) was proposed for rapid identification of infected patients at high risk of death.^[13]^ The qSOFA criteria is very similar to CRB and consists of the same 3 clinical parameters, however the cut-off for hypotension (systolic blood pressure ≤100 mm Hg versus diastolic blood pressure ≤60 mm Hg or systolic blood pressure <90 mm Hg in CRB) and tachpnea (respiratory rate>21/min vs >29/min in CRB) were different.^[13,14]^ The definition group recommended using a qSOFA score ≥2 plus a suspected infection to help identify patients with potential sepsis outside of the intensive care unit (ICU), and a qSOFA score ≥2 provided validity for mortality prediction as good as that of the SOFA score for patients with suspected infection in non-ICU. However, as pneumonia is a major source of sepsis, it is important to assess the outcome prediction abilities of the qSOFA criteria in patients with pneumonia. In addition, it is also necessary to compare the qSOFA criteria to other pneumonia-specific scores for outcome prediction.

In the present study, we performed a meta-analysis to evaluate the predictive performance of the qSOFA criteria in patients with pneumonia.

## 2 Materials and methods

This study was performed according to the Preferred Reporting Items for Systematic Reviews and Meta-Analyses (PRISMA) statement.

### 2.1 Search strategy and selection criteria

We systematically searched Embase, Google Scholar, and PubMed (up to March 2018) by using the following strategy: (“qSOFA” or “quick-SOFA” or “quick sequential organ failure assessment”) and (“respiratory tract infection” or “respiratory infection” or “pneumonia”). To ensure a comprehensive literature search, we examined reference lists from included studies. In this meta-analysis, we included studies based on the following criteria: prospective or retrospective studies, a clear diagnostic reference standard for pneumonia was used, the aim was to assess the prognostic value of qSOFA score in predicting mortality in patients with pneumonia, and eligible studies should have adequate information to construct a 2 × 2 contingency table (true positives [TP], false positives [FP], false negatives [FN], and true negatives [TN]). The excluded criteria were as follows: studies which had only an abstract, review articles, letters, expert opinions, and conference abstracts. All articles were evaluated independently by 2 investigators (JJ and JY); any disagreements were resolved by group consensus.

### 2.2 Data extraction and quality assessment

The descriptive data were extracted as follows: first author, country of origin, year of publication, study design, clinical setting, endpoint, mortality, sample size, TP, FP, FN, and TN. If any items that required clarification, we contacted the corresponding author by emails. To assess quality, modified criteria according to the criteria of Hayden et al^[15]^ were used. We assessed the following 6 items: population, follow-up, measurement of severity scores, outcome measurement, confounding variables, and statistical analysis. Each item was scored from 0 to 2. When publications had scores ≥11, they were graded as the high-quality ones.

### 2.3 Ethical statement

All analyses and results were from previous published studies; thus, no patient consent and ethical approval are required.

### 2.4 Statistical analysis

Statistical analysis was conducted by the Meta-Disc 1.4 (XI Cochrane Colloquium; Barcelona, Spain) and STATA 11.0 software (Stata Corporation, college station, TX). We tabulated TP, FN, FP, and TN according to the effects of qSOFA (<2 and ≥2) on mortality in patients with pneumonia. RR (Relative Risk) was used to assess the predict value of qSOFA, which was pooled by fixed-effects or random-effects models based on the DerSimonian and Laird's method.^[16]^*I*^2^ index and *Q*-test were used to assess heterogeneity.^[17,18]^*I*^2^ value > 50% represent a significant level of heterogeneity and the random-effects model was chosen. Otherwise, the fixed-effects model was used.^[16]^

The pooled sensitivity, specificity, diagnostic odds ratio (DOR), positive likelihood ratio (PLR), negative likelihood ratio (NLR) were calculated using a bivariate random-effects regression model.^[19,20]^ A summary receiver operating characteristic curve (SROC) was generated to evaluate the overall diagnostic accuracy.^[21]^ We conducted a subgroup analysis to explore the primary source of heterogeneity and assess the prognostic accuracy of the qSOFA when studies were restricted to emergency department (ED) setting, CAP patients, retrospective studies, and prospective studies only.

## 3 Results

**Figure 1:**
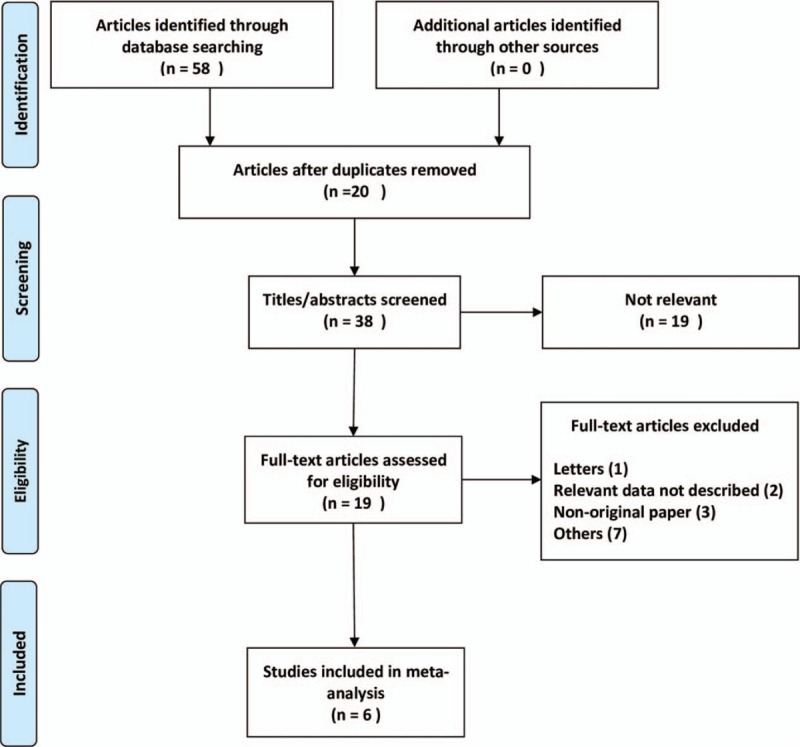
Flow diagram of the study selection process.

Our database search retrieved 58 articles, based on the exclusion and inclusion criteria, 52 articles were excluded. A total of 6 studies^[22–27]^ met the inclusion criteria in our meta-analysis, one^[24]^ of which included 2 trials. Therefore, a total of 7 trials with 17,868 cases were included (Fig. 1). No articles have been identified by searching for references.

### 3.1 Study characteristics

Table 1 revealed the main characteristics of the included studies. The 6 included articles were published between 2016 and 2017. These studies principally originated from Europe (one from Germany,^[22]^ Spain^[24]^ and Switzerland^[26]^ respectively), and Asia (one from China,^[25]^ Korea,^[23]^ and Japan^[27]^ respectively). All the studies were published in English. Two studies^[22,24]^ were described as prospective, and 4 studies^[23,25–27]^ were described as retrospective. Four studies^[23–26]^ were done in ED. The majority of the studies used 30-day mortality or 28-day mortality as their primary outcome measure, and the mortality rates varied from 3% to 33%.

**Table 1:**
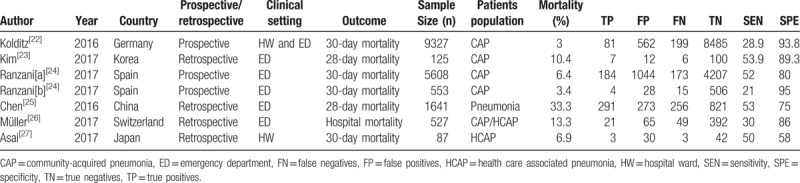
Characteristics of included studies.

### 3.2 Quality assessment

We performed a quality assessment based on the criteria developed by Hayden et al,^[15]^ 4 studies were considered moderate (9 to 10); and 2 good (≥11) quality (Table 2).

**Table 2:**
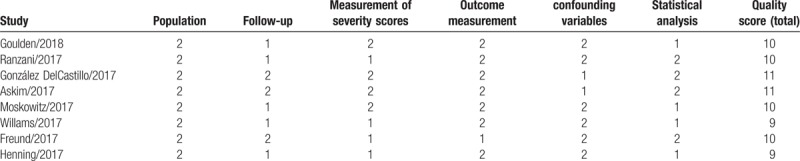
Study quality assessment.

### 3.3 Predictive value of a qSOFA score ≥2 on short-term death in patients with pneumonia

**Figure 2:**
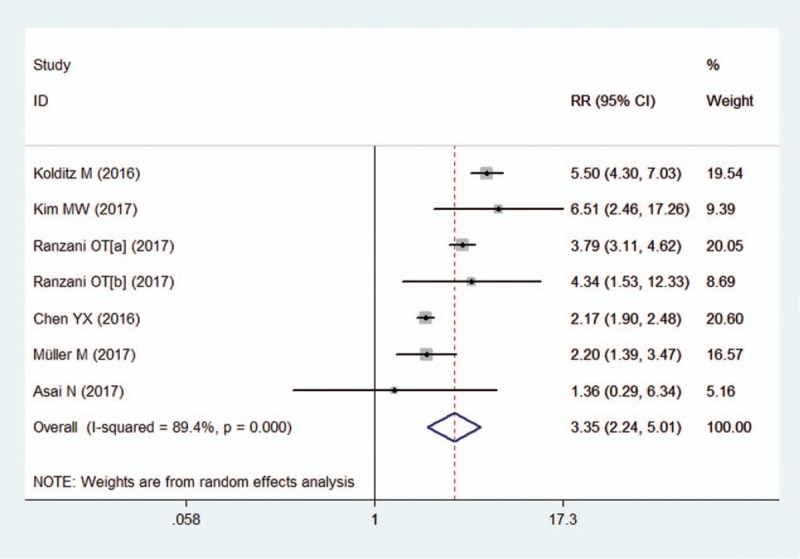
Forest plot of qSOFA score ≥2 to predict mortality in pneumonia. qSOFA = quick sequential organ failure assessment.

All the included studies showed that a qSOFA score ≥2 was related to a higher risk for death in patients with pneumonia, with RR ranging from 1.36 to 6.51. Due to the significant heterogeneity between the studies, we used a random-effects model to pooled RR estimates. The pooled RR was 3.35 (95% CI, 2.24–5.01) (Fig. 2).

**Figure 3:**
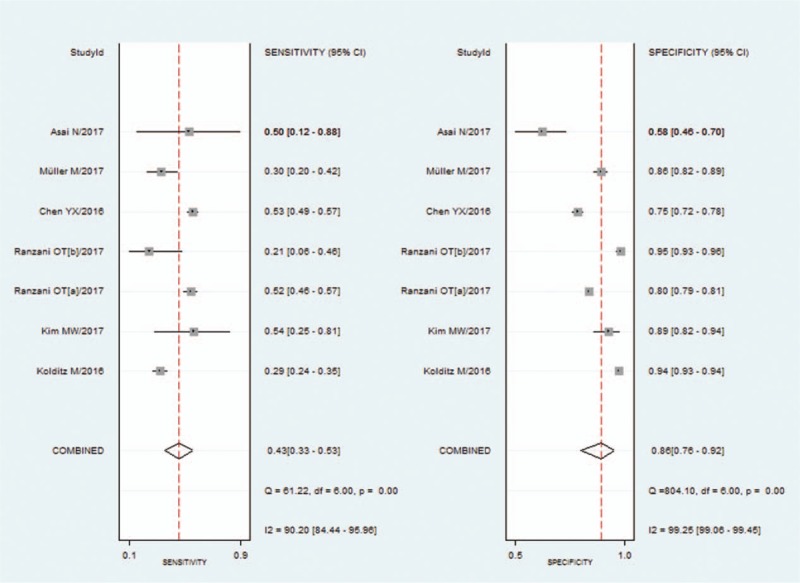
Forest plot of the sensitivity and specificity of qSOFA score ≥2 for predicting mortality in pneumonia. qSOFA = quick sequential organ failure assessment.

**Figure 4:**
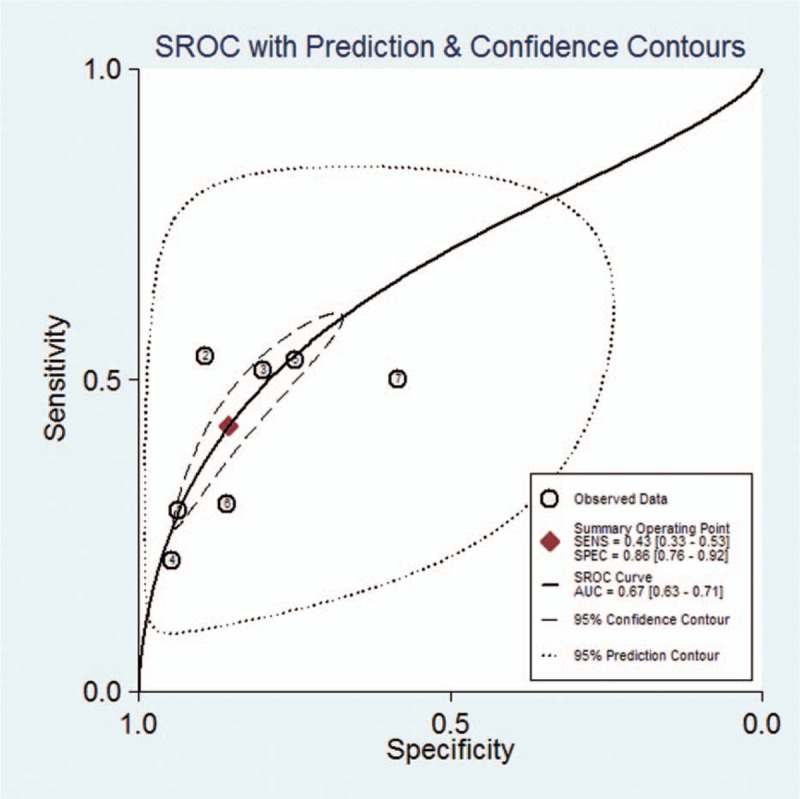
Summary receiver operating characteristic graph of the included studies.

We used a bivariate random-effects regression model to conduct the diagnostic test accuracy meta-analysis and to assess the overall sensitivity and specificity of a qSOFA score ≥2 in predicting death of pneumonia. The pooled sensitivity and specificity were 0.43 (95% CI, 0.33–0.53) and 0.86 (95% CI, 0.76–0.92), respectively (Fig. 3). The PLR and NLR were 3.0 (95% CI, 2.2–4.0) and 0.67 (95% CI, 0.61–0.74), respectively. The diagnostic OR was 4 (95% CI, 3–6). The area under the SROC curve was 0.67 (95% CI, 0.63–0.71) (Fig. 4).

### 3.4 Subgroup analysis

A subgroup analysis restricted ED setting, CAP patients, retrospective studies, and prospective studies were performed (Table 3). It was found that none of the subgroup analysis influenced the main conclusions.

**Table 3:**
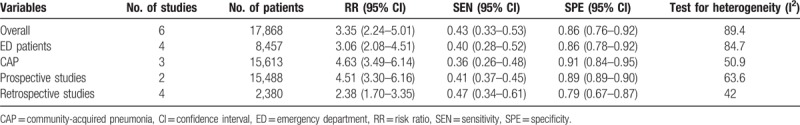
Subgroup analysis.

### 3.5 Sensitivity analysis and publication bias

To evaluate the stability to the conclusion of this meta-analysis, we conducted a sensitivity analysis by dropping each study sequentially, and the sensitivity analysis indicated that the results of the meta-analysis were robust. Due to the limited number of studies available, we did not assess publication bias in this meta-analysis.

## 4 Discussion

Pneumonia is a major source of sepsis and a common cause of mortality. For critically ill patients, the uncertain outcomes and unpredictable course of the disease is still a challenge for clinicians, hindering the early recognition of patients at risk of death. Early identification of these patients is paramount to ensure early initiation of the appropriate therapeutic interventions and ultimately to improve patient outcomes‘. In this meta-analysis, we first determined that a qSOFA score ≥2 was associated with moderate prognosis in patients with pneumonia. This finding suggests that qSOFA score may be used for risk stratification, as well as prognosis of a pneumonia.

The qSOFA criteria are very similar to CRB and contain 3 identical vital signs: blood pressure, respiratory rate, and mentation. However, the criteria thresholds of hypotension and tachpnea were stricter for CRB than for qSOFA; Assessment of altered mentation by qSOFA was simpler than CRB. Therefore, it is necessary to compare the qSOFA criteria to other ‘traditional’ scoring systems for outcome prediction. Kolditz et al^[22]^ showed that the prognostic accuracy of the qSOFA was similar to the CRB for in-hospital mortality among adults with pneumonia; Yet specificity is lower and sensitivity higher. Chen et al^[25]^ found that patients with a qSOFA score ≥2 had higher mortality than patients with same CRB-65 score. They further concluded that qSOFA was better than CRB-65 in distinguishing pneumonia patients with high risk for death. Müller et al^[26]^ reported that a qSOFA score ≥2 was related to a higher risk for death in pneumonia, and they also revealed that the qSOFA and CURB-65 scores were equal in their predictive ability of in-hospital mortality in patients with pneumonia. However, in respect to predict ICU admissions, qSOFA is superior to CURB-65. However, the comparison the predictive performance of the qSOFA with other ‘traditional’ scoring systems remains controversial. Ranzani et al^[24]^ demonstrated that the predictive performance of the CRB and CURB-65 was better than qSOFA for CAP patients. Due to the limited number of studies available, we could not perform head-to-head comparison of qSOFA score and other “traditional” scoring systems for the prediction of mortality in patients with pneumonia. In this meta-analysis, we demonstrated that a qSOFA score ≥2 was related to a higher risk of death in patients with pneumonia, with pooled RR was 3.35 (95% CI, 2.24–5.01), suggesting that a qSOFA score ≥2 predict moderate prognosis for a pneumonia.

We further explore the prognostic performance of the qSOFA score. In terms of predicting mortality, the high specificity of qSOFA is valuable for screening pneumonia patients who are more likely to have adverse outcomes, so the qSOFA score can be used to prompt clinicians to further check the presence of organ dysfunction in pneumonia patients, to start or escalate appropriate therapy, or to consider referring patients to the ICU. However, the poor sensitivity of qSOFA score means that some patients at higher risk for death may be misclassified and managed as nonserious patients.

There were several limitations in this meta-analysis. First, the main limitation is the limited number of studies included, which may not completely evaluate the prognostic potential of qSOFA score. Second, this meta-analysis indicated significant heterogeneity among the included studies. The studies’ included CAP patients and HCAP patients, and different outcome measures were used, such as hospital mortality, 28-day mortality or 30-day mortality. Pneumonia patients in different clinical settings were observed in the studies, including emergency pneumonia patients and ward pneumonia patients, and different designs were used in the studies, including retrospective and prospective observational studies. Despite these variations, comprehensive subanalyses indicated conclusions similar to those of the main analysis. Conclusions were not influenced by considering separately those studies including different type of pneumonia, studies using different designs and studies including different clinical setting. These analyses have significantly improved homogeneity while not affecting the main conclusions. However, despite the multiple subanalyses performed, the meta-analysis was still influenced by biases inherent in the included studies. Some researchers proposed that there was often a significant heterogeneity in systematic reviews of diagnostic studies.^[28]^ Third, due to the limited number of studies available, we could not perform head-to-head comparison of qSOFA score and other “traditional” scoring systems for the prediction of mortality in patients with pneumonia.

## 5 Conclusions

In summary, a qSOFA score ≥2 was strongly associated with mortality in patients with pneumonia, and a qSOFA score ≥2 had prognostic significance in assessing the mortality of pneumonia in adult patients. Since no laboratory testing is required, qSOFA appears to be a rapid, effective, and simple way to identify patients at high risk of death. But the poor sensitivity of qSOFA may have limitations in the early identification of mortality in patients with pneumonia, so it seems necessary to find ways to improve its low sensitivity.

## Author contributions

**Conceptualization:** Jianjun Jiang, Jin Yang, Yongmei Jin, Youjin Lu.

**Data curation:** Jianjun Jiang, Yongmei Jin.

**Formal analysis:** Jianjun Jiang.

**Funding acquisition:** Jin Yang.

**Investigation:** Jianjun Jiang, Jin Yang.

**Methodology:** Jianjun Jiang, Yongmei Jin.

**Project administration:** Jianjun Jiang, Youjin Lu.

**Resources:** Jianjun Jiang, Yongmei Jin.

**Software:** Jianjun Jiang, Yongmei Jin, Jiyu Cao.

**Supervision:** Jiyu Cao, Youjin Lu.

**Validation:** Jin Yang, Youjin Lu.

**Visualization:** Jin Yang, Youjin Lu.

**Writing – original draft:** Jianjun Jiang.

**Writing – review & editing:** Jin Yang, Youjin Lu.
